# The effect of image fractal properties and its interaction with visual discomfort on gait kinematics

**DOI:** 10.1038/s41598-023-42114-0

**Published:** 2023-10-03

**Authors:** D. Burtan, J. F. Burn, B. Spehar, U. Leonards

**Affiliations:** 1 School of Psychological Science, University of Bristol, Bristol, UK; 2 Queen’s School of Engineering, University of Bristol, Bristol, UK; 3 School of Psychology, UNSW Sydney, Sydney, NSW Australia

**Keywords:** Human behaviour, Cognitive neuroscience

## Abstract

Exposure to images of urban environments affords higher cognitive processing demands than exposure to images of nature scenes; an effect potentially related to differences in low-level image statistics such as fractals. The aim of the current study was to investigate whether the fractal dimensions of an abstract scene affect cognitive processing demands, using gait kinematics as a measure of cognitive demand. Participants (n = 40) were asked to walk towards different types of synthetic images which were parametrically varied in their fractal dimensions. At the end of each walk, participants rated each image for its visual discomfort (n = 20) or for its likability (n = 20) as potential confounding factors. Fractal dimensions were predictors of walking speed. Moreover, the interaction between fractal dimensions and subjective visual discomfort but not liking predicted velocity. Overall, these data suggest that fractal dimensions indeed contribute to environmentally induced cognitive processing demands.

## Introduction

It is well established in psychological research that exposure to nature environments as compared to urban environments has positive effects on cognitive functioning^1^,e.g.^2–5^). This is thought to be related to nature environments not including as many distractions as urban environments, thus not requiring the same amount of attention and therefore not depleting mental resources in the same way^6,7^. This effect, known as the *nature benefit*, is expressed in higher cognitive/attentional task performance after prolonged exposure to nature environments than to urban environments, and comparably lower stress levels^8–10^. Even exposure to visual information only (e.g. photographs) can have similar effects (e.g.^8^), suggesting that at least some of the positive effects of exposure to nature environments are driven by visual aspects of the environment. Yet, whether these visual aspects are related to low-level or high-level sensory processing (e.g.^11^) or to factors such as aesthetics^12^, visual discomfort (e.g.^13^), memories or scene-related semantics^14^, remains unclear and a matter of increased research focus^15–17^.

Using changes in basic gait kinematics, such as step length and velocity, as a measure of cognitive load, we have recently shown that the impact of environment type (nature vs. urban) on cognitive processing load does not require prolonged exposure to the respective environments but can be picked up over several seconds exposure on a trial-by-trial basis^18,19^: in line with expectations for increased cognitive load (see for a review^20^), participants walked more slowly and with shorter steps toward urban scenes than toward nature scenes. This opens a new promising way to investigate individual visual aspects for their contribution to increased cognitive load for urban scenes or decreased cognitive load for nature scenes^8,9^. (Please note that we are only interested in relative differences in cognitive load between environments, and not whether any differences found are due to a nature benefit or to an urban cost^12^).

Our approach to measure gait kinematics as an indicator of cognitive load is based on four assumptions (see also^21^): First, each person has a preferred walking speed and stride length to reduce the metabolic cost of ambulation^22^, leading to a very stable gait pattern when walking over flat, obstacle-free ground with a maximum within-person variability of around 2.3% for speed and 2.0% for stride length, respectively (e.g^23^). Second, the visual system has evolved in animals to help them guide their actions through and interactions with the environment around them (^24,25^, for an excellent review on vision and action see^26^). Any *systematic* change in speed induced by sensory changes of the environment is thus notable and meaningful. Third, due to evolutionary selection pressures, the sensitivity of our visual system has become optimised to goal-relevant signals which includes more efficient processing of information prevalent but irrelevant for survival within the environmental niche of our ancestors^27^. And fourth, similar to other factors increasing cognitive load, an increase in environmentally-induced perceptual load slows a person’s preferred gait speed and step length, irrespective of the person’s gender or age^20,28^.

In line with the third assumption above, the Perceptual Fluency Account by Joye and colleagues (PFA;^11,29,30^) suggests that much of the low-level visual demands on cognitive processing load of urban environments relates to a lack of fluent perceptual processing (i.e. a lack of efficient processing for task-irrelevant perceptual information outside the environmental niche of our ancestors). That nature scenes are processed more quickly than urban scenes might thus be a by-product of the fluent perceptual processing inherent in their low-level sensory features; i.e. their basic image statistics such as their increased fractal dimensions. Indeed, participants were able to perform complex tasks more easily after exposure to images with higher fractal dimensions typical for nature scenes than after exposure to images with lower fractal dimensions typical for urban scenes^11^.

Fractals are defined as patterns whose structural complexity is repeated multiple times across different spatial scales; the repeating pattern is identical in the so-called exact or mathematical fractals, while many natural forms and patterns exhibit statistical similarity across different spatial scales^31^. Fractal dimensions of outdoor visual scenes have been proposed as an indicator for environmentally induced cognitive load, with first indications that this might affect walking speed^28^. The fractal properties of natural environments and scenes are also related to the scale-invariant statistics revealed in their spatial frequency regularities, in particular in their 1/f amplitude spectra with an alpha range of 0.8–1.5 and a mean of 1.2 (e.g.^32^), corresponding to a range of fractal dimensions (D) between 1.50 and 1.65. Moreover, sensitivity thresholds in the human fovea and parafovea have been found to be lowest for 1/f amplitude spectra with alphas between 1.2 and 1.4 (e.g.^33^) which is in line with our third assumption that such information is processed more efficiently due to its (task-irrelevant) prevalence in the environmental niche of our ancestors (i.e. nature)^27^.

The aim of the current study was therefore to investigate whether the fractal dimension of an image affects cognitive processing demands, using gait kinematics as the measure of demand. We hypothesised that walking towards images with fractal properties outside the range typically found in nature scenes should result in a decrease in participants’ walking speed, smaller step length and an increase in stride time, in line with findings of gait changes for higher cognitive load (e.g.^18,20,28,34^).

**Figure 1:**
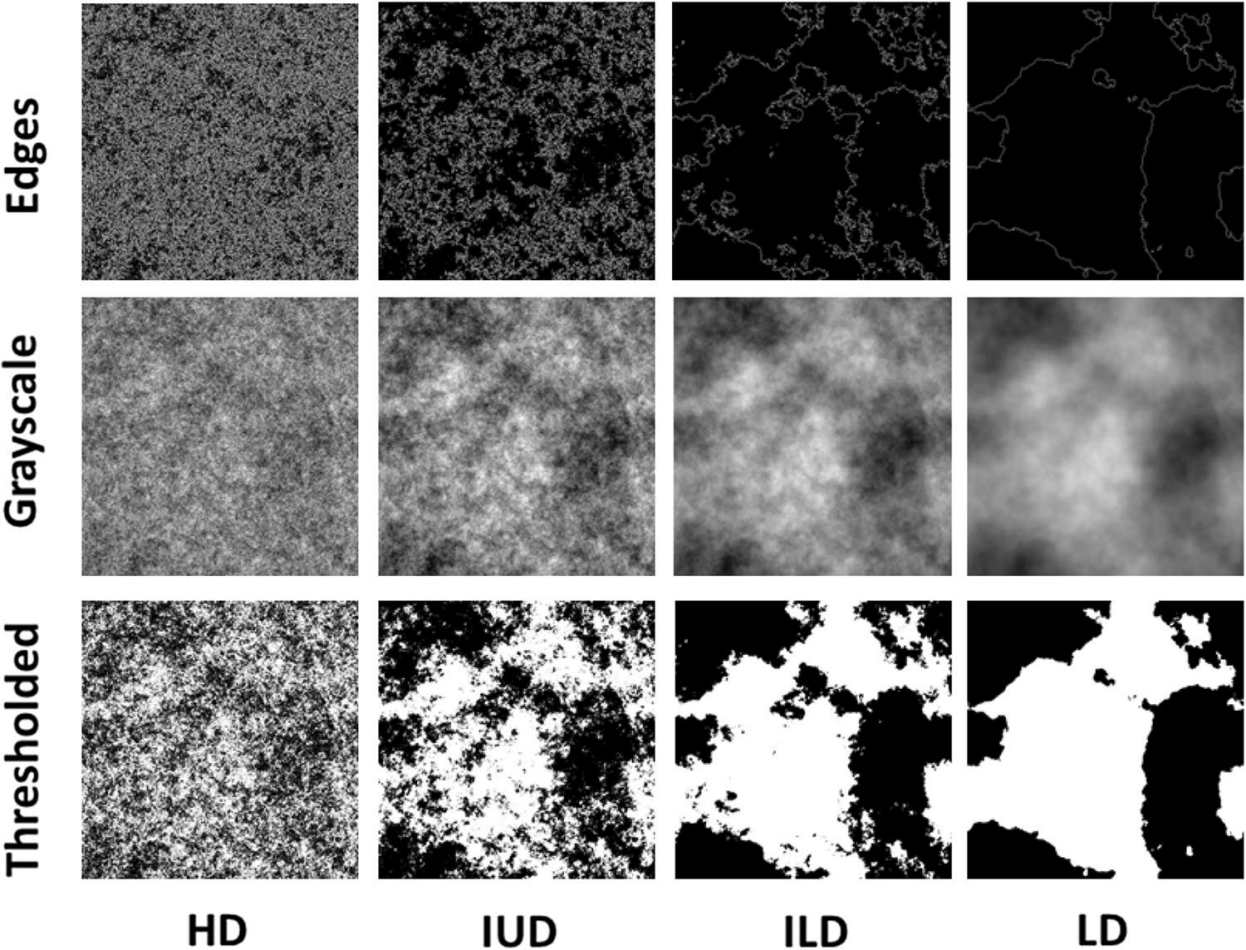
Example of selected abstract images which were parametrically varied in their fractal dimension/D value; and from the right to left: High Dimension (HD) with amplitude spectrum slopes (alpha) of 0.8 and fractal dimensions between 1.75 and 1.90, Intermediate Upper Dimension (IUD) with amplitude spectrum slopes (alpha) of 1.2 and fractal dimensions between 1.50–1.65, Intermediate Lower Dimension (ILD) with amplitude spectrum slopes (alpha) of 1.6 and fractal dimensions between 1.25 and 1.40, and Low Dimension (LD) with amplitude spectrum slopes (alpha) of 2.0 and fractal dimensions between 1.0 and 1.15. Image types were Edges, Grayscale and Thresholded (from top to bottom).

 To test this hypothesis, participants (n = 40) were asked to walk towards images that were parametrically varied in their fractal dimensions around an intermediate fractal scaling range typical for nature images, whilst their gait was recorded using 3D motion capture. As fractals can vary substantially in their visual appearance without differing in their fractal-like scaling and geometric properties^35^, participants were presented with three different types of synthetic fractal images (Edges, Grayscale and Thresholded, see Fig. 1; for a detailed description of image types, see^35^ and^31^) with similar fractal properties to ensure that any changes in gait would generalise across image types. Further, to control for any stimulus material induced changes in aesthetic preference or visual discomfort that had been reported before to affect changes in gait kinematics^18,19^, participants were asked to rate each image for its visual discomfort (n = 20) or its likeability (n = 20).

## Results

Statistical analysis, including multi-level modelling, followed the same analysis steps as detailed in our previous publications^18,19^.

Repeated measures MANOVAs, 2 (cognitive rating task) × 4 (fractal dimension) × 3 (image type), were conducted on the following gait measures: mean velocity, mean step length, and mean stride time. (Note that this analysis did not include the control condition; i.e. the condition without fractal images that served exclusively as a reference to the literature for participants’ preferred walking speed and step length walking over flat obstacle free ground without additional task).

### Velocity

**Figure 2:**
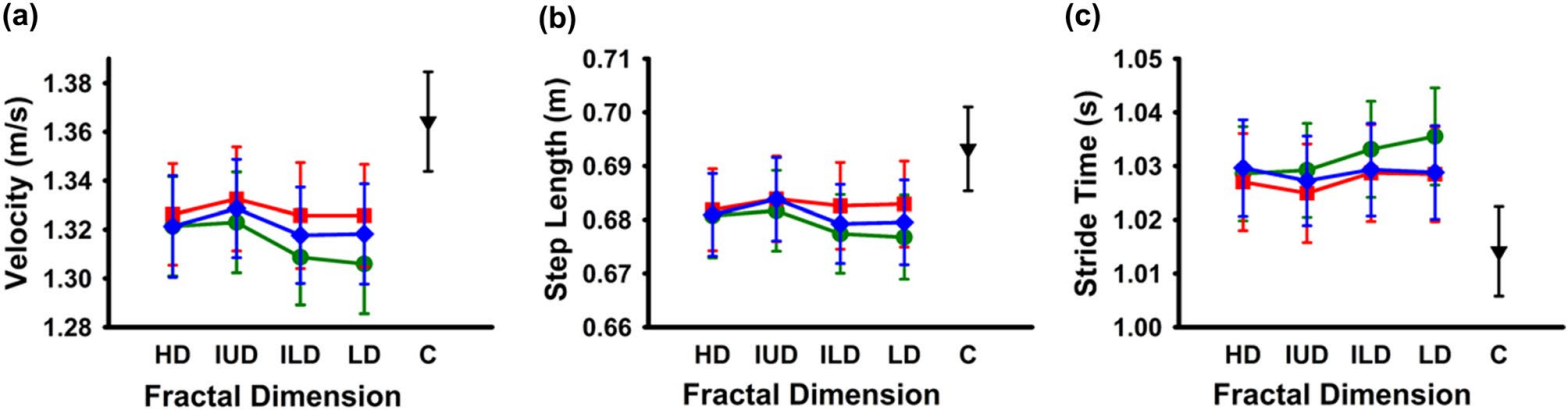
Group averages (n = 39) of (**a**) individual mean velocity (m/s), (**b**) individual mean step length (in meters) and (**c**) individual mean stride time (in seconds) across fractal dimensions: HD (High D:1.75–1.90), IUD (Intermediate Upper D: 1.50–1.65), ILD (Intermediate Lower D: 1.25–1.40), and LD (Low D: 1.0–1.15) for the three image types (Edges—green circles; Grayscale—red squares, Thresholded—blue rhombi). Error bars reflect ± 1 SEM. Group averages for the respective control conditions (**c**) are shown for comparison as black triangles.

There was a statistically significant main effect of *fractal dimension* on velocity determined by MANOVA with Greenhouse–Geisser correction, F(2.374, 1.483) = 8.012, *p* < 0.001, partial η^2^ = 0.178 (see Fig. 2a). *Post-hoc* pairwise comparisons using Bonferroni corrections revealed that the Intermediate Upper Dimension (IUD) condition had a significantly faster walking speed than both Intermediate Lower Dimension (ILD; *p* < 0.05) and Low Dimension (LD; *p* < 0.001) conditions. There was no significant difference between High Dimension (HD) and IUD conditions, nor between HD and ILD or LD conditions (*p* > 0.05); (see Methods Section for further details about the four ranges of fractal dimensions).

Moreover, there was a statistically significant main effect of *image type* on velocity determined by MANOVA with Greenhouse–Geisser correction, F(1.483, 54.861) = 8.500, *p* < 0.05), partial η^2^ = 0.187. *Post-hoc* pairwise comparisons using Bonferroni correction revealed that participants walked significantly slower when presented with Edges only images than both Grayscale (*p* < 0.05) or Thresholded (*p* < 0.05) images.

### Step length

There was a statistically significant main effect of fractal dimension on step length, F (3,111) = 7.813, *p* < 0.001, partial η^2^ = 0.174 (see Fig. 2b). *Post-hoc* pairwise comparisons using Bonferroni correction revealed that the IUD condition had a significantly longer step length (*p* < 0.05) than any of the other conditions.

Further, there was a statistically significant main effect of image type on step length, F(2, 74) = 8.892, *p* < 0.05, partial η^2^ = 0.194. *Post-hoc* pairwise comparisons using Bonferroni correction revealed that participants walked with significantly shorter steps toward Edges only images as compared to both Grayscale (*p* < 0.05) or Thresholded (*p* < 0.05) images.

### Stride time

As for velocity and step length, there was also a statistically significant main effect of fractal dimension on stride time determined by MANOVA with Greenhouse–Geisser correction, F(2.274, 57.211), *p* < 0.001, partial η^2^ = 0.121 (see Fig. 2c). *Post-hoc* pairwise comparisons using Bonferroni correction revealed that the IUD condition led to significantly shorter stride times than the LD condition (*p* < 0.05); but there were no significant differences between any other comparisons (*p* > 0.05).

There was a statistically significant main effect of image type on stride time determined by MANOVA with Greenhouse–Geisser correction, F(1.546, 57.211) = 5.511, *p* < 0.05, partial η^2^ = 0.130. *Post-hoc* pairwise comparisons using Bonferroni correction revealed that participants had significantly longer stride times during exposure to Edges only images as compared to exposure to both Grayscale (*p* < 0.05) and Thresholded images (*p* < 0.05).

Cognitive task (visual discomfort rating task vs. liking rating task) did not differentially affect any of the gait measures, nor were there any significant interactions between task and fractal dimensions, task and image type, or fractal dimensions and image type for any of the gait measures.

### Rating tasks

#### Visual discomfort

**Figure 3:**
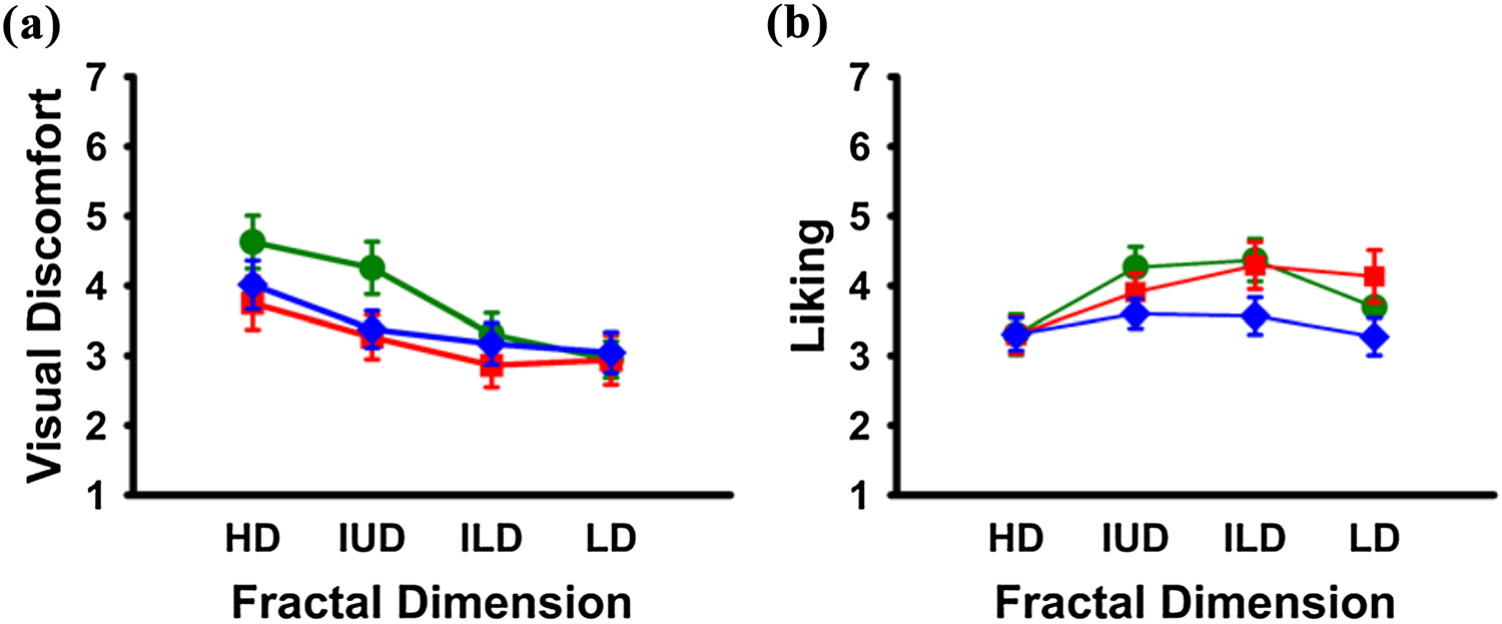
Group averages of (**a**) visual discomfort (n = 20; left panel) and (**b**) liking (n = 19; right panel) across fractal dimensions: HD (High D: 1.75–1.90), IUD (Intermediate Upper D: 1.50–1.65), ILD (Intermediate Lower D: 1.25–1.40), and LD (Low D: 1.0–1.15)] for three image types (Edges—green circles, Grayscale—red squares, Thresholded—blue rhombi). Error bars reflect ± 1 SEM.

As can be seen in Fig. 3a, visual discomfort decreased with a decrease in fractal dimensions and was particularly high for the HD and IUD images of the Edges only type. This is reflected in the outcomes of a repeated measures ANOVA with Greenhouse–Geisser correction on visual discomfort ratings (n = 20) as dependent measure, and fractal dimension [HD, IUD, ILD, LD] and image type as independent factors (E, G, T). There was a statistically significant main effect of fractal dimension on visual discomfort, F(1.265, 24.027) = 16.018, partial η^2^ = 0.427, *p* < 0.001. *Post-hoc* pairwise comparisons using Bonferroni correction revealed that the HD condition had a significantly higher visual discomfort score than IUD, ILD and LD conditions (*p* < 0.05). Moreover, IUD had a significantly higher visual discomfort score than ILD and LD conditions (*p* < 0.05). There was no significant difference between ILD and LD conditions (*p* > 0.05).

Crucially, whilst image type per se did not impact visual discomfort ratings (*p* > 0.05), there was a statistically significant interaction between fractal dimension and image type on visual discomfort, F(3.551, 67.474) = 5.076, partial η^2^ = 0.211, *p* < 0.05). *Post-hoc* pairwise comparisons using Bonferroni correction revealed that the HD-E condition led to significantly higher visual discomfort scores than the corresponding HD-G and HD-T conditions (*p* < 0.05). Similarly, the IUD-E condition had a significantly higher visual discomfort score than the IUD-T condition. Visual discomfort ratings for ILD and LD conditions, in contrast, did not differ between image types.

#### Liking

Liking ratings seemed to follow an inverted U-shape for different fractal dimensions as can be seen in Fig. 3b. This was confirmed by a repeated measures ANOVA with Greenhouse Geisser correction on liking scores (n = 19) as the dependent measure, and fractal dimension [HD, IUD, ILD, LD] and image type (E, G, T) as independent measures. There was a statistically significant main effect of fractal dimension on liking, F(1.483,26.876) = 1.472, partial η2 = 0.252, *p* < 0.05. *Post-hoc* pairwise comparisons using Bonferroni correction revealed that the HD condition had a significantly lower liking score than IUD (*p* < 0.05) and ILD (*p* < 0.05) conditions. Also, the ILD condition had a significantly higher liking score than the LD Condition (*p* < 0.05). None of the other comparisons were significant.

Image type did not affect liking scores but, as for visual discomfort, there was a statistically significant interaction between fractal dimension and image type. *Post-hoc* pairwise comparisons using Bonferroni correction revealed, however, that none of the meaningful comparisons reached significance.

### Multi-level modelling

Whilst the MANOVAs performed above allowed us to observe basic effects of fractal dimensions on gait kinematics, such analyses do not control for the impact of image variability and individual discomfort or aesthetic preference ratings nor their possible interaction with changes in fractal dimensions on gait kinematics.

As already alluded to at the beginning of the results section, we therefore applied multi-level modelling to cross-classified data from both cognitive tasks (n = 39) to determine the impact of fractal dimensions, image type, and task on gait velocity (m/s), using MLwiN 3.03 software (for an in-depth description of the procedures used here see earlier studies^18,19^). In short, we fitted a cross-classified model using the Markov chain Monte Carlo (MCMC) method with the Bayesian Deviance Information Criterion (DIC) as is suggested to handle more complex cross-classified models^36^. Analysis was restricted to velocity for comparison with earlier studies^18,19^ in which velocity had been the most sensitive of the three gait measures.

Again, data for control images were excluded from this analysis, not least due to missing data for fractal dimension and image type. Velocity data were transformed into Z-scores.

The multi-level structure treats all trials (N = 3703) as nested within both image and participant. Three variables were classified as random effects, with trial at level 1, and image and participant at level 2 (model 1, see Supplementary material Table S2).

A series of models was fitted to establish the model of best fit, using the lowest Deviance Information Criterion (DIC) statistics as selection criterion (see Supplementary material Table S2).

The best fitting model included fractal dimension ($X_{1}^{2}$ = 7.022) and image type ($X_{1}^{2}$ = 2.231), both being significant predictors (*p* < 0.05) for velocity, in line with the results of our earlier MANOVA (see Supplementary material, Table S2). Parameter estimates for the model are displayed in Table 1.

**Table 1:** Fixed effects estimates (top) and random effect variance estimates (bottom) for the model with the best fit (Model 2a; see Supplementary material, Table S2).

Parameter	Estimate	Std. error	95% CI lower	95% CI upper	$X_{1}^{2}$
**Fixed**					
Intercept	0.035	0.191	-0.334	0.391	0.879
Fractal dimension	− 0.020	0.008	-0.035	-0.005	7.022*
image type	0.023	0.01	0.003	0.044	2.231*
**Random**					
Participant	0.900	0.217	0.569	1.413	
Image	0.002	0.001	0.001	0.004	
Trial	0.180	0.004	0.172	0.189	
Deviance information criterion (DIC)					4237.487

Estimates reflect the size of the effect on standardised velocity. Burn-in = 500, Chain Length = 10,000. Degrees of freedom is 1 for all Chi-square ($X_{1}^{2}$) statistics. *p < 0.05.

#### Visual discomfort

To investigate the potential confounding impact of subjective visual discomfort on walking speed, we performed a second multilevel modelling analysis, focusing on cross-classified velocity data of the visual discomfort rating group only (n = 20). Again, control images were excluded from this analysis due to missing data for fractal dimension and image type. Both velocity and visual discomfort rating data were transformed into Z-scores.

The multilevel structure treats all trials (N = 1887) as nested within both image and participant. Three variables were classified as random effects, with trial at level 1, image and participant at level 2 (model 1, see Supplementary material Table S3).

The results of this analysis revealed that the best fitting model included both, fractal dimension ($X_{1}^{2}$ = 6.853) and its interaction with standardised subjective visual discomfort ($X_{1}^{2}$ = 32.388) were significant predictors, *p* < 0.05. Note that, in contrast to our MANOVA, the interaction between image type and fractal dimension was not a significant predictor for walking speed; also, please note that Visual Discomfort on its own did not predict walking speed.

Parameter estimates for the model are displayed in Table 2.

**Table 2:** Fixed effects estimates (top) and random effect variance estimates (bottom) for the model with best fit (Model 3a; see Supplementary material, Table S3).

Parameter	Estimate	Std. error	95% CI lower	95% CI upper	$X_{1}^{2}$
**Fixed**					
Intercept	0.058	0.243	− 0.427	0.539	
Fractal dimension	− 0.032	0.012	− 0.056	− 0.008	6.853*
Fractal dimension * visual discomfort	− 0.031	0.005	− 0.041	− 0.020	32.388**
**Random**					
Participant	0.883	0.331	0.455	1.706	
Image	0.004	0.002	0.001	0.009	
Trial	0.239	0.008	0.224	0.256	
Deviance Information criterion (DIC)					2703.025

Estimates reflect the size of the effect on standardised velocity. Burn-in = 500, Chain Length = 10,000. Degrees of freedom is 1 for all Chi-square ($X_{1}^{2}$) statistics. **p < 0.001, *p < 0.05.

#### Liking

To investigate the potential confounding impact of subjective liking on walking speed, we performed an additional multilevel-modelling analysis on the cross-classified and Z-scored velocity data of the likeability rating group only (n = 19), with likeability Z-scored. Control images were again excluded.

The multilevel structure treats all trials (N = 1816) as nested within both image and participant. Three variables were classified as random effects, with trial at level 1, image and participant at level 2 (model 1, see Supplementary material Table S4). Note that the data from the likeability rating task suggested a nonlinear relationship between fractal dimensions and liking (see Fig. 3b); therefore, liking-squared was included as an additional predictor variable.

The results of this analysis revealed that the best fitting model included fractal dimension as the only significant predictor ($X_{1}^{2}$ = 8.366, p < 0.05) for walking speed (see Supplementary material, Table S4). Please note that this differs from our findings for visual discomfort ratings.

Parameter estimates for the model are displayed in Table 3.

**Table 3:** Fixed effects estimates (top) and random effect variance estimates (bottom) for the model with best fit (Model 2a; see Supplementary material, Table S4).

Parameter	Estimate	Std. error	95% CI lower	95% CI upper	$X_{1}^{2}$
**Fixed**					
Intercept	− 0.015	0.164	− 0.358	0.276	0.008
Fractal dimension	− 0.026	0.009	− 0.044	− 0.008	8.366*
**Random**					
Participant	0.997	0.369	0.516	1.913	
Image	0.002	0.001	0.001	0.005	
Trial	0.141	0.005	0.132	0.151	
Deviance information criterion (DIC)					1639.064

Estimates reflect the size of the effect on standardised velocity. Burn-in = 500, Chain Length = 10,000. Degrees of freedom is 1 for all Chi-square ($X_{1}^{2}$) statistics. *p < 0.05.

## Discussion

The present study provides support for our hypothesis that the fractal dimension of an image affects a person’s gait kinematics which we used here as a proxy measure of cognitive load (see^18,20^): walking speed and step length increased and stride time decreased with increasing fractal dimensions from a fractal scaling range of 1.0 to 1.65. Thus, walking towards images with fractal properties outside the range typically found in nature scenes^32^ (1.0–1.15), seemed more cognitively demanding than walking towards images with fractal dimensions within this range (1.5–1.65). Interestingly, our data also indicate that not only fractal dimensions but also image type impacts cognitive load: walking towards Edges only images resulted in a decrease in participants’ walking velocity, and smaller step length as compared to walking towards Thres holded and Grayscale images, suggesting that Edges only stimuli were more cognitively demanding despite having the same fractal dimensions as their Thres holded and Grayscale counterparts. It is important to note, however, that the interpretation of data for Edges only stimuli is complicated by the fact that, unlike with the Grayscale and Thres holded stimuli, their mean luminance does not remain constant across fractal dimensions. Both the mean luminance and the standard deviation of luminance values are much higher for HD and IUD images than for ILD and LD images (see Supplementary material, Table S1), making the latter abstract patterns potentially harder to visually discern and rate for liking and/or visual discomfort. Moreover, stimuli lowered the overall luminance within the laboratory which might further have affected gait due to an increased uncertainty about the visual environment. These decreases in walking speed related to different image properties are observed on top of participants’ general task-related slowing as compared to the speed of natural self-paced walking without exposure to images and related rating task (i.e. our control condition), a slowing effect in line with the gait literature on dual tasking (see for a review^20^).

Together, these findings thus seem to support Joye and colleagues’ Perceptual Fluency Account (PFA;^11,29,30^) of nature scenes being less cognitively demanding than urban scenes due to their low-level visual features; in particular, as tested here, their fractal dimension.

Before drawing any firm conclusions, however, there are some caveats to consider.

Firstly, although statistically highly significant, one might feel that the difference in walking speed between different fractal dimension conditions was comparably small, with a variability in average velocity across conditions of 0.04 m/s (i.e. between 1.3 and 1.34 m/s). However, when seen in the context of our first and second assumptions for this study, namely that a person’s preferred walking speed and step length are highly stable whilst walking over flat obstacle-free ground with a maximum within-person variability of around 2.3% for speed (e.g.^23^) and that the visual system has evolved to guide safely human movements through their environments (e.g.^21,26^), a within-person *systematic* slowing of around 3% following changes in fractal dimensions of images covering only a small area of the visual field is notable and physiologically meaningful.

Secondly, we need to ascertain that instead of fractal dimension, changes in rating difficulty across the different fractal and stimulus conditions could not explain the observed gait changes. We think such an interpretation unlikely as gait kinematics did not differ between likeability rating and visual discomfort rating groups. Moreover, the likeability group revealed the same kind of gait changes for changes in fractal dimension as the visual discomfort group although liking itself was an insignificant predictor of walking speed; nor was there an interaction between likeability scores and fractal dimension. Thus, neither an image’s likeability nor its interaction with low level image statistics seemed to contribute to environmentally induced cognitive load. Whilst dual tasking thus generally affects gait kinematics for all conditions compared to self-paced natural walking speed without cognitive task or without image content (compare gait kinematics between all testing conditions and our control condition in Fig. 2), neither the observed change in gait kinematics for different fractal dimensions nor the interaction between fractal dimensions and visual discomfort ratings can be explained by changes in task difficulty. Nevertheless, to independently confirm cognitive load differences for different fractal dimensions, future studies might want to use different experimental measures for cognitive load such as task-irrelevant attentional capture (see also^18^).

Thirdly, an interaction between fractal dimension and visual discomfort ratings predicting changes in gait speed for our multi-level analysis (visual discomfort group, n = 20) seems at first glance in line with previous findings for natural images^18^: the higher the subjective visual discomfort of an environment, the slower a person walks toward it. A closer look at this interaction, however, speaks against such an interpretation: participants rated not only images with high fractal dimensions as the most uncomfortable ones, but also images with intermediate upper fractal dimensions thought to fall right into the range of fractal dimensions typical for nature scenes. This rating behaviour contrasts with participants’ gait kinematics, where participants walked the fastest toward intermediate upper fractal dimensions as one would predict for reduced perceptual load for image statistics within the range of fractal dimensions typical for nature. What might have induced this discrepancy between visual discomfort ratings and gait kinematics will have to be answered in future experiments.

Against expectations, participants rated images with intermediate upper fractal dimensions as typical for nature scenes as more uncomfortable to look at than images with low and intermediate lower fractal dimensions. Aesthetics did not explain this effect as neither fractal dimensions nor interactions between fractal dimensions and image type affected liking scores. It is more likely that the interaction between fractal dimensions and image type played a role in this effect. Edges only images with high fractal dimensions had higher subjective visual discomfort ratings than Grayscale and Thresholded images. Similarly, Edges only images with high fractal dimensions had higher subjective visual discomfort ratings than Thresholded images for the intermediate upper condition.

Last but not least, it is important to consider whether there might be alternative interpretations to changes in cognitive load for the observed gait slowing. In particular, one of our reviewers wondered whether our data could be explained by embodied or situated cognition models independently of cognitive load. As highlighted in the assumptions we made in the introduction on the evolution of the visual system and its role in guiding locomotion, and based on the particular experimental design we chose, any systematic slowing of gait is related to differences in stimulus processing evoked by different fractal dimensions. As such, gait differences can be easily explained with Bayesian decision making models in the Vision for Action literature which are supported by ample neuroscientific evidence (see in particular^26^): processing fractal dimensions outside the range found in nature is equivalent to increasing visual noise in the environment (for review see^21^). With increased noise, visual processing is less efficient, thus increasing uncertainty about the visual state of the world and ultimately hampering the visual system to safely guide our actions. To reduce this uncertainty and with it the risk of high locomotor costs (e.g. a trip or a fall), gait is slowed and decision making for the next foot placement delayed. That this perceptual load can be equated to cognitive load through its reliance on decision making processes can be seen by the interaction with visual discomfort, further slowing gait. Alternative interpretations such as embodied cognition models based on Gibson’s idea of affordances as a measure of what an environment offers an animal to support its actions^37^ would have to assume different affordances for different fractal dimensions. To the best of our knowledge, there is to-date no neuroscientific evidence for the existence of such affordances (for an excellent review on the distinction between vision for action and affordances, see^38^).

In conclusion, walking towards images with fractal properties outside the range typically found in nature scenes (D = 1.50–1.65) slows gait and thus seems more cognitively demanding in line with Joye’s Perceptual Fluency Account^11,29,30^ and our third assumption that our visual system is optimised to process goal-relevant signals of environments from our evolutionary past^27^. However, our data also provide evidence for a more complex interaction between low-level image statistics such as fractal dimensions and visual discomfort ratings that contributes to environmentally induced cognitive load. It is tempting to speculate what might underlie this latter interaction. If one accepts that visual discomfort is an indicator of physiological stress^39^, then this would align with Ulrich’s Stress Recovery Theory^10^ that physiological stress contributes to environmentally induced cognitive load measurable as changes in gait kinematics. Visual discomfort is a subjective measure, not an objective measure of stress. Future studies might therefore want to test the prediction above, using physiological measures of stress such as blood pressure, heart rate or the level of the stress hormone cortisol see^40^ together with gait analysis.

## Methods

### Participants

Based on effect sizes observed in our earlier studies^18,19^, forty participants (33 females, 7 males, mean age = 20 ± 3.02 SD years, aged between 18–33 years) took part in this study. They were randomly assigned to one of two groups: twenty participants to the visual discomfort rating group (16 females, 4 males, mean age = 21 years + − 3.95 SD, aged between 18 and 33) and the other twenty participants (17 females, 3 males, mean age = 19 years + − 3.95 SD, aged between 18 and 24 years) to the likeability rating group. Note that gender had not been controlled for as meta-analyses of many studies indicate that sex differences in preferred walking speed are small and statistically and physiologically insignificant, even in the presence of significant variation in height and body mass (for review see^41^). Moreover, as already mentioned in the introduction, cognitive load has been shown to affect gait irrespective of gender or age^20,28^.

Prior to their experimental session, participants were provided with both verbal and written information about the study, including information about breaks and their right to withdraw from the study at any time. Participants reported normal or corrected-to-normal visual acuity and no neurological conditions that could affect their walking. Moreover, they reported good physical health and were aware that they would have to walk for about an hour for this experiment. All participants signed a consent form, were debriefed at the end of the experiment, and received compensation for their time in form of course credit.

The experiment was approved by the Faculty of Life Sciences’ Ethics Committee at the University of Bristol (ref. 10,101,994,923).

### Stimuli

The stimulus set contained 105 synthetic images: 96 images were parametrically varied in their fractal dimension /D value see^42^), and 9 were plain gray images serving as control stimuli.

Fractal dimensions fell into four ranges:

1. High Dimension (HD 1.75–1.90) with amplitude spectrum slopes (alpha) of 0.8;
2. Intermediate Upper Dimension (IUD 1.50–1.65) with amplitude spectrum slopes (alpha) of 1.2;
3. Intermediate Lower Dimension (ILD 1.25–1.40) with amplitude spectrum slopes (alpha) of 1.6;
4. Low Dimension (LD 1.0–1.15) with amplitude spectrum slopes (alpha) of 2.

To increase the variability in visual appearance without changing fractal-like scaling and geometric properties, each fractal dimension was presented in three different image types Grayscale, Thresholded, and Edges (see Fig. 1), developed and studied before by Spehar and colleagues^31,35^ Selecting three different image types ensured that any observable effects of fractal dimension were not specific to the particular stimulus material chosen. Note that Grayscale images contain two-dimensional fractals (i.e. fractal variations are related to surface-texture appearances) whilst their Thresholded (black and white) and Edges only counterparts contain one-dimensional fractals (i.e. fractal properties are determined by variations in fractal contours).

Following the methods described in^31^ and^35^ in more detail, we first created grayscale images in Matlab by producing a 512 × 512 grid of random pixels (with values between 0 and 255) selected from a Gaussian distribution. A Fast Fourier transform was then performed to create a series of amplitude spectra at four desired levels of α falloff (α = 0.8, 1.2, 1.6 and 2.0). An inverse Fourier transform applied each amplitude spectrum to the 512 × 512 Gaussian noise image, resulting in images possessing specific desired α values. The Thresholded image variants were created by bisecting the Grayscale images at the mean luminance value and converting all pixels below and above the mean luminance to black and white respectively. Finally, the Edges only images were created from the Thresholded images by an edge extraction procedure, resulting in solid light lines on a dark background for the Edges only image variations. For each of the input amplitude spectrum slopes of the Grayscale images, the respective root mean square (RMS) contrast, mean luminance, measured fractal dimension and amplitude spectrum slope values of the resulting images were calculated (see Supplementary material, Table S1).

Therefore, the 96 fractal images were categorised into twelve image conditions, with 8 images per condition:

- HD: Edges (8), HD: Grayscale (8), HD: Thresholded (8);
- IUD: Edges (8), IUD: Grayscale (8), IUD: Thresholded (8);
- ILD: Edges (8), ILD: Grayscale (8), ILD: Thresholded (8);
- LD: Edges (8), LD: Grayscale (8), LD: Thresholded (8).

Image resolution of fractal images was 800 × 800 pixels.

### Task and procedure

The same task and procedure were used as described in detail in our previous studies^18,19^.

In brief, participants were asked to wear a belt at hip height with three small spherical retro-reflective markers attached to detect the left hip, right hip, and lower abdomen (hereon jointly referred to as hip). Markers were also attached to participants’ shoulders (lateral clavicle), knees (patella), the outside of their ankles (lateral malleolus), and to their feet (first metatarsal-phalangeal joint). A 3D motion capture system (Oqus, Qualisys AB, Sweden) was used to detect these markers during walking. The system consisted of 12 cameras (x-direction depicting lateral movement; y-direction depicting direction of forward movement; z-direction depicting vertical movement), capturing 12 m x 2 m × 2.4 m of laboratory space. The system was calibrated prior to the start of each experimental session to provide an accuracy of 1mm^3^ for the reflective markers. The recording frequency was 100 Hz.

The participants’ task was to walk repeatedly down the laboratory (15 m) in their naturally preferred walking speed and in the straightest possible way toward the images projected onto the back wall by an Optoma EW536 projector, one image per walk (i.e. trial). Expected starting and end points for each walk were marked on the floor. During each trial, one of the 105 images described above was projected in a random order. The image display size was 2 m wide x 2 m high, corresponding to 7.6° × 7.6° of visual angle at the start line and 38° × 38° of visual angle at the end line of the 3D motion capture space).

After each walk and before returning back to the starting position for the next experimental trial, half of the participants were asked to rate verbally the image just seen for visual discomfort (How uncomfortable is this image to view?) on a 7-point Likert Scale from 1 = ‘extremely comfortable to view’ to 7 = ‘extremely uncomfortable to view’ (visual discomfort rating group); the other half of the participants rated images verbally for likability (How much do you like the image?) on a 7-point Likert Scale from 1 = ‘not at all’ to 7 = ‘very much’ (likability rating group). Participants’ responses were recorded by the experimenter.

In addition, we introduced 9 “control” trials, randomly intermixed with experimental trials, to establish participants’ preferred gait kinematics in laboratory settings without dual tasking (i.e. without rating the images after the walk) to serve as a reference to the existing gait literature. These trials used plain gray images without fractal dimensions.

There were two planned breaks during the session (after trials 35 and 70, respectively), and participants had been made aware that they could ask for additional breaks if required. None of the participants required additional breaks. The task took approximately 60 min to complete.

Automatic Identification of Markers was applied to the 3D motion capture raw data, in particular foot and hip markers in proprietary software (Qualisys Track Manager (QTM), Qualisys AB). The procedure of step detection and trial removal due to QTM missing data has been described in detail in earlier studies, see^18,19^. To base analysis on data with constant walking speed only, data were truncated to the middle of the run. Mean velocity (walking speed), step length, and stride time, respectively, were calculated for each trial by subtracting position of foot and hip markers (Y-position), and calculating lifting and landing time of each foot, see^18^.

One participant was excluded from the analysis due to a loss of sensor data during testing. This left 19 (2 male) participants’ datasets for analysis for the likeability group, aged 18–24 years (M = 19, SD = 1.53). Across the two task conditions, there was thus a total of 39 participants’ datasets included in the analysis (7 male), aged between 18 and 33 years (M = 19, SD = 3.06). As confirmed with our control condition (i.e. walking over a flat obstacle free ground without task and without fractal dimensions), participants’ preferred walking speed lay on average at 1.36 m/s, in line with earlier studies in the same laboratory^18,19^ and slightly below the clinical norms reported in the literature for young healthy adults of 1.4 m/s^43^.

### Supplementary Information

Supplementary Tables.

## Data Availability

Materials and Data are available at the University of Bristol data repository, data.bris, at https://doi.org/10.5523/bris.u36u3g8uq0372ii1wpc583gxv.

## References

[1] Kaplan S, Berman MG (2010). Directed attention as a common resource for executive functioning and self-regulation. Perspect. Psychol. Sci..

[2] Cimprich B, Ronis DL (2003). An environmental intervention to restore attention in women with newly diagnosed breast cancer. Cancer Nurs..

[3] Hartig T, Evans GW, Jamner LD, Davis DS, Gärling T (2003). Tracking restoration in natural and urban field settings. J. Environ. Psychol..

[4] Ottosson J, Grahn P (2005). A comparison of leisure time spent in a garden with leisure time spent indoors: on measures of restoration in residents in geriatric care. Landsc. Res..

[5] Tennessen CM, Cimprich B (1995). Views to nature: Effects on attention. J. Environ. Psychol..

[6] Kaplan S (1995). The restorative benefits of nature: Toward an integrative framework. J. Environ. Psychol..

[7] Kaplan R (2001). The nature of the view from home. Environ. Behav..

[8] Berman MG, Jonides J, Kaplan S (2008). The cognitive benefits of interacting with nature. Psychol. Sci..

[9] Berman MG (2012). Interacting with nature improves cognition and affect for individuals with depression. J. Affect. Disord..

[10] Ulrich RS (1984). View through a window may influence recovery from surgery. Science.

[11] Joye Y, Steg L, Unal AB, Pals R (2016). When complex is easy on the mind: Internal repetition of visual information in complex objects is a source of perceptual fluency. J. Exp. Psychol. Hum. Percept. Perform..

[12] Bratman GN, Hamilton JP, Daily GC (2012). The impacts of nature experience on human cognitive function and mental health. Ann. N. Y. Acad. Sci..

[13] Penacchio O, Wilkins AJ (2015). Visual discomfort and the spatial distribution of Fourier energy. Vis. Res..

[14] Vo ML, Boettcher SE, Draschkow D (2019). Reading scenes: How scene grammar guides attention and aids perception in real-world environments. Curr. Opin. Psychol..

[15] Twedt E, Rainey RM, Proffitt DR (2019). Beyond nature: The roles of visual appeal and individual differences in perceived restorative potential. J. Environ. Psychol..

[16] Meidenbauer KL (2019). The gradual development of the preference for natural environments. J. Environ. Psychol..

[17] Meidenbauer KL (2020). The affective benefits of nature exposure: What's nature got to do with it?. J. Environ. Psychol..

[18] Burtan D (2021). The nature effect in motion: visual exposure to environmental scenes impacts cognitive load and human gait kinematics. R. Soc. Open Sci..

[19] Burtan D, Burn JF, Leonards U (2021). Nature benefits revisited: Differences in gait kinematics between nature and urban images disappear when image types are controlled for likeability. PLoS ONE.

[20] Amboni M, Barone P, Hausdorff JM (2013). Cognitive contributions to gait and falls: evidence and implications. Mov. Disord..

[21] Leonards UB (2023). A Sense of Plausibility in Vision and Music Perception.

[22] Bertram JE (2005). Constrained optimization in human walking: cost minimization and gait plasticity. J. Exp. Biol..

[23] Collins SH, Kuo AD (2013). Two independent contributions to step variability during over-ground human walking. PLoS ONE.

[24] Goodale MA, Humphrey GK (1998). The objects of action and perception. Cognition.

[25] Bridgeman B, Tseng P (2011). Embodied cognition and the perception-action link. Phys. Life Rev..

[26] Hayhoe MM (2017). Vision and action. Annu. Rev. Vis. Sci..

[27] Endler JA (1992). Signals, signal conditions, and the direction of evolution. Univ. Chicago Press.

[28] Ho S, Mohtadi A, Daud K, Leonards U, Handy TC (2019). Using smartphone accelerometry to assess the relationship between cognitive load and gait dynamics during outdoor walking. Sci. Rep..

[29] Joye Y, Van den Berg A (2011). Is love for green in our genes? A critical analysis of evolutionary assumptions in restorative environments research. Urban For. Urban Green..

[30] Joye Y, De Block A (2011). 'Nature and I are Two': A critical examination of the Biophilia hypothesis. Environ. Values.

[31] Spehar B, Clifford CWG, Newell BR, Taylor RP (2003). Universal aesthetic of fractals. Comput. Graph..

[32] Tolhurst DJ, Tadmor Y, Chao T (1992). Amplitude spectra of natural images. Ophthalmic Physiol. Opt..

[33] Hansen BC, Hess RF (2006). Discrimination of amplitude spectrum slope in the fovea and parafovea and the local amplitude distributions of natural scene imagery. J. Vis..

[34] Patel P, Lamar M, Bhatt T (2014). Effect of type of cognitive task and walking speed on cognitive-motor interference during dual-task walking. Neuroscience.

[35] Spehar B, Walker N, Taylor RP (2016). Taxonomy of Individual variations in aesthetic responses to fractal patterns. Front. Hum. Neurosci..

[36] Kass RE, Carlin BP, Gelman A, Neal RM (1998). Markov chain Monte Carlo in practice: A roundtable discussion. Am. Stat..

[37] Gibson JJ (1979). The Ecological Approach to Visual Perception.

[38] Ferretti G (2021). A distinction concerning vision-for-action and affordance perception. Conscious Cogn..

[39] Wilkins AJ (1984). A neurological basis for visual discomfort. Brain.

[40] Crosswell AD, Lockwood KG (2020). Best practices for stress measurement: How to measure psychological stress in health research. Health Psychol. Open.

[41] Bruening DA, Frimenko RE, Goodyear CD, Bowden DR, Fullenkamp AM (2015). Sex differences in whole body gait kinematics at preferred speeds. Gait Posture.

[42] Viengkham C, Spehar B (2018). Preference for fractal-scaling properties across synthetic noise images and artworks. Front. Psychol..

[43] Perry J, Garrett M, Gronley JK, Mulroy SJ (1995). Classification of walking handicap in the stroke population. Stroke.

