# Supplementary material for: The effect of image fractal properties and its interaction with visual discomfort on gait kinematics
Source: Sci Rep. 2023 Oct 3;13:16581. doi: 10.1038/s41598-023-42114-0 (PMC10547763; doi:10.1038/s41598-023-42114-0)
Supplement: Supplementary file 1 — Supplementary Tables. [file 41598_2023_42114_MOESM1_ESM.docx]

**Supplementary Materials**

**Title:** The effect of image fractal properties and its interaction with visual discomfort on gait kinematics

**Authors:** Burtan, Burn, Spehar and Leonards

**Table S1.** Measured image properties.

|  | | RMS Contrast | | Luminance | | FD | | ASS | |
| --- | --- | --- | --- | --- | --- | --- | --- | --- | --- |
|  |  | M | SD | M | SD | M | SD | M | SD |
| Edges | | | | | | | | | |
|  | HD | 118.9 | 0.66 | 81.51 | 1.69 | 2.06 | 0.01 | -0.41 | 0.02 |
|  | IUD | 81.24 | 4.93 | 29.42 | 3.98 | 1.73 | 0.04 | -0.63 | 0.03 |
|  | ILD | 36.34 | 5.68 | 5.42 | 1.66 | 1.33 | 0.06 | -0.6 | 0.04 |
|  | LD | 18.12 | 2.35 | 1.31 | 0.35 | 1.07 | 0.03 | -0.52 | 0.02 |
| Grayscale | | | | | | | | | |
|  | HD | 38.20 | 0.00 | 127.50 | 0.00 | 1.94 | 0.01 | -0.79 | 0.01 |
|  | IUD | 38.20 | 0.00 | 127.50 | 0.01 | 1.62 | 0.04 | -1.17 | 0.03 |
|  | ILD | 38.20 | 0.01 | 127.50 | 0.00 | 1.22 | 0.06 | -1.6 | 0.08 |
|  | LD | 38.20 | 0.00 | 127.50 | 0.00 | 0.93 | 0.03 | -2.04 | 0.16 |
| Thresholded | | | | | | | | | |
|  | HD | 127.50 | 0 | 127.57 | 0.25 | 1.94 | 0.01 | -0.69 | 0.01 |
|  | IUD | 127.03 | 0.07 | 128.78 | 4.76 | 1.62 | 0.04 | -1.03 | 0.04 |
|  | ILD | 127.40 | 0.34 | 130.89 | 11.09 | 1.22 | 0.06 | -1.31 | 0.13 |
|  | LD | 126.70 | 0.66 | 131.15 | 14.02 | 0.93 | 0.03 | -1.49 | 0.10 |

Note. SD was calculated of all pixels, RMS = Root mean square, FD = Fractal Dimension, ASS = Amplitude Spectrum Slope, HD = High D, IUL = Intermediate Upper D, ILD = Intermediate Lower D, LD = Low D. Fractal dimensions of images had been calculated by applying the box-counting technique (see ^1^.) Note that this box-counting technique is a comparably rough estimate of fractal dimensions and subject to errors arising from arbitrary grid placement known as quantization error (see ^2^).

**Table S2.** Model fit comparisons for models with standardised velocity as a dependent variable. fitted; models lettered ‘a’ show the best combination of predictors at each stage, following the removal of insignificant predictors.

| **Model** | **DIC** | **Fixed** | **Random** |
| --- | --- | --- | --- |
| 1 | 4242.919 |  | PT, IM, T |
| 2 | 4237.634 | FD, IT, T | PT, IM, T |
| 2a | 4237.487 | FD, IT | PT, IM, T |
| 3 | 4239.068 | FD, IT, FD*IT, FD*T, IT*T | PT, IM, T |
| 3a | 4242.919 | Model 1 | PT, IM, T |

Random effects: PT = Participant, IM = Image, T=Trial. Fixed effects: FD = Fractal Dimension, IT = Image type, T = Task.

**Table S3.** Model fit comparisons for models with standardised velocity as a dependent variable; visual discomfort group (N=1887). Models lettered ‘a’ show the best combination of predictors at each stage, following the discarding of insignificant predictors.

| **Model** | **DIC** | **Fixed** | **Random** |
| --- | --- | --- | --- |
| 1 | 2732.676 |  | PT, IM, T |
| 2 | 2705.245 | FD, IT, VD | PT, IM, T |
| 2a | 2706.540 | FD, VD | PT, IM, T |
| 3 | 2703.640 | FD, VD, FR*VD, FR*IT, VD*IT | PT, IM, T |
| 3a | 2703.025 | FD, FD*VD | PT, IM, T |

Random effects: PT = Participant, IM = Image, T=Trial. Fixed effects: FD = Fractal Dimension, IT = Image type, VD = Visual Discomfort.

**Table S4**. Model fit comparisons for models with standardised velocity as a dependent variable; likeability group (N=1816). Models lettered ‘a’ show the best combination of predictors at each stage, following the discarding of insignificant predictors.

| **Model** | **DIC** | **Fixed** | **Random** |
| --- | --- | --- | --- |
| 1 | 1644.237 |  | PT, IM, T |
| 2 | 1643.738 | FD, IT, LIK, LIK^2 | PT, IM, T |
| 2a | 1639.064 | FD | PT, IM, T |
| 3 | 1642.242 | FD, FD*IT, FD*LIK, FD*LIK^2, IT*LIK, IT^LIK2 | PT, IM, T |
| 3a | 1640.101 | FD,FD*IT,IT*LIK^2 | PT, IM, T |

Random effects: PT = Participant, IM = Image, T=Trial. Fixed effects: FD = Fractal Dimension, IT = Image type, LIK = Liking.

1 Viengkham, C. & Spehar, B. Preference for Fractal-Scaling Properties Across Synthetic Noise Images and Artworks. *Frontiers in Psychology* **9**, doi:10.3389/fpsyg.2018.01439 (2018).

2 Bouda, M., Caplan, J. S. & Saiers, J. E. Box-Counting Dimension Revisited: Presenting an Efficient Method of Minimizing Quantization Error and an Assessment of the Self-Similarity of Structural Root Systems. *Front Plant Sci* **7**, 149, doi:10.3389/fpls.2016.00149 (2016).
